# Brain Drain for Brain Gain: Potential Applications of Robotic-assisted Lymphatic Microsurgery in the Management of Neurological Disorders

**DOI:** 10.1097/GOX.0000000000007191

**Published:** 2025-10-03

**Authors:** Jennifer A. Watson, Samuel Knoedler, Donata von Reibnitz, Carmen E. Zurfluh, Carlotta Imholz, Giuseppe Esposito, Simon J. Schreiner, Epameinondas Gousopoulos, Aijia Cai, Sedef Kollarik, Pietro Giovanoli, Christian Baumann, Nicole Lindenblatt

**Affiliations:** From the * Department of Plastic Surgery and Hand Surgery, University Hospital Zurich, Zurich, Switzerland; † Department of Neurosurgery, University Hospital Zurich, Zurich, Switzerland; ‡ Department of Neurology, University Hospital Zurich, Zurich, Switzerland; § Faculty of Medicine, University of Zurich, Zurich, Switzerland.

## Abstract

**Background:**

The central nervous system (CNS) was long believed to be devoid of lymphatic drainage. However, the discovery of the glymphatic system and meningeal lymphatics has revolutionized our understanding of cerebrospinal fluid homeostasis and neuroimmune interactions. The glymphatic system facilitates perivascular cerebrospinal fluid–interstitial fluid exchange and promotes neurotoxic waste clearance, whereas meningeal lymphatics serve as conduits between the CNS and peripheral lymphatic circulation. Dysfunction in these lymphatic efflux pathways has been implicated in the pathogenesis of neurological disorders such as Alzheimer disease, Parkinson disease, traumatic brain injury, and intracranial hemorrhage, where impaired waste removal contributes to protein aggregation, neuroinflammation, and hence, disease onset and progression.

**Methods:**

Recent preliminary evidence suggests that surgical modulation of lymphatic drainage may offer novel therapeutic avenues for these disorders, with lymphatic microsurgery, particularly deep cervical lymphovenous anastomosis (LVA), proposed as an innovative procedure to enhance CNS lymphatic outflow. The first case reports in Alzheimer disease patients demonstrated not only the operative feasibility of LVA but also postoperative cognitive improvements. Despite these promising findings, systematic (pre)clinical studies remain scarce, calling for further research.

**Results:**

This article examined the role of the brain lymphatic system in neurological disorders and discussed the potential of lymphatic microsurgery as a novel therapeutic intervention. We also highlight ongoing clinical trials and potential future innovations, including surgical robotic assistance, and report on 2 cases of deep neck LVA for central lymphatic disorders.

**Conclusions:**

By combining neurolymphatic research with surgical advances, LVAs have the potential to redefine therapeutic paradigms in CNS disorder management.

**Takeaways**

**Question:** How does cerebral lymphatic drainage dysfunction contribute to neurological disorders, and can lymphatic microsurgery play a role in reconstructing lymphatic flow?

**Findings:** Emerging preclinical evidence suggests that deep cervical lymphovenous anastomoses may enhance brain waste clearance. Robotic-assisted lymphatic reconstruction may aid in advancing the field in the future.

**Meaning:** Enhancing CNS lymphatic drainage via lymphatic microsurgery represents a promising therapeutic avenue for the treatment of neurodegenerative diseases.

## INTRODUCTION

The interplay between the central nervous system (CNS) and the lymphatic system has long been poorly understood. Historically, the brain was thought to be immune-privileged, lacking conventional lymphatic drainage. Histological evidence of lymphatic vessels could not be identified in the brain parenchyma, in part because of postmortem changes in the tissue. The blood-brain barrier was believed to prevent the diffusion of molecular and cellular components. However, the discovery of functional meningeal lymphatic vessels and the glymphatic system has redefined our understanding of CNS fluid dynamics, implicating lymphatic function in neurological health and disease.^1^ The glymphatic system, a perivascular network facilitating cerebrospinal fluid (CSF) and interstitial fluid (ISF) exchange, is postulated to play a key role in neurotoxic waste clearance, with meningeal lymphatics serving as conduits linking the CNS and peripheral lymphatic circulation.^2^ These findings have sparked interest in the contribution of lymphatic dysfunction to the pathogenesis and etiology of neurological disorders.^1,3–5^

The notion of “brain drain” via microsurgical optimization of cerebral lymphatic outflow may herald a new era in CNS disorder therapy; as reflected in the article’s title, this concept proposes that promoting lymphatic clearance from the brain (“drain”) could result in functional neurological improvement (“gain”).^6,7^ Deep cervical lymphovenous anastomoses (LVAs), connecting deep cervical lymphatic vessels to veins, could enhance toxic protein aggregate clearance, reduce neuroinflammation, and influence the trajectory of neurological disorders.^7–9^ This article investigates whether lymphatic microsurgery—specifically deep cervical LVA—can serve as a novel therapeutic strategy for enhancing CNS lymphatic clearance in neurological disorders, with a particular focus on the role of robotic-assisted techniques in enabling the precision, feasibility, and broader clinical translation of this emerging approach.

## LYMPHATIC DRAINAGE SYSTEM OF THE BRAIN

### Glymphatic System

For a long time, the brain’s toxic metabolite removal was assumed to rely solely on intracellular degradation and slow diffusion across the blood-brain barrier.^2,10^ However, recent discoveries have identified a previously unrecognized fluid clearance pathway—the glymphatic system—enabling rapid and efficient exchange between CSF and ISF.^1^ Coined by the neuroscientist Nedergaard, “glymphatic” reflects the system’s dependence on glial cells, particularly astrocytes, and its functional similarity to the peripheral lymphatic system.^2^

Approximately 200 mL of CSF circulates within the brain, with complete turnover occurring roughly 4 times daily.^11^ Although some is quickly reabsorbed into the bloodstream upon reaching the subarachnoid space, another portion flows along leptomeningeal perivascular spaces (Virchow–Robin spaces) lined by astrocyte endfeet that facilitate rapid fluid movement.^1,2^ Arterial pulsations, vasomotor tone, and inspiratory pressure fluctuations potentially modulate these perivascular spaces, circulating CSF into the brain.^12,13^ Astrocytic endfeet surround the blood vessels entering the brain and are populated with water-selective aquaporin-4 (AQP4) channels, which enable rapid water molecule movement.^1^ As CSF flows through the interstitial compartment, it collects metabolic waste products, such as beta-amyloid (Aβ), tau proteins, and other neurotoxic substances. Sleep plays a critical role in this machinery, facilitating the brain’s nightly clearance of toxic metabolites.^14^ The link between sleep and clearance was first demonstrated in live mice by Xie et al,^15^ who reported a 60% increase in interstitial space during sleep accompanied by an increase in glymphatic flow and Aβ clearance.

### Meningeal Lymphatics

Predominantly situated in the dura mater along the dural sinuses and the skull base, the meningeal lymphatic vessels provide a direct route for CSF drainage from the intracranial glymphatic to the peripheral lymphatic system. The meningeal lymphatic network seems to be located near the eyes, following a path above the olfactory bulb before settling alongside the sinuses and cranial nerves, and ultimately, exiting the cranium through foramina along with their respective structures. The meningeal lymphatic network is made up of narrower vessels.^10^ These vessels collect CSF along with its solute components, including metabolic waste products and immune cells, after the fluid has circulated along the glymphatic pathway from the subarachnoid space via arachnoid granulations. Following the intracerebroventricular injection of fluorescent tracer dye into anesthetized adult mice, Louveau et al^10^ validated the functional capability of the meningeal lymphatic vessels to transport CSF and their topographic alignment along the superior sagittal sinus. Draining CSF into the peripheral lymphatic system, the meningeal lymphatics facilitate the clearance of potentially harmful proteins and metabolic waste from the CNS.

### Cervical Lymph Nodes

**Fig. 1.**
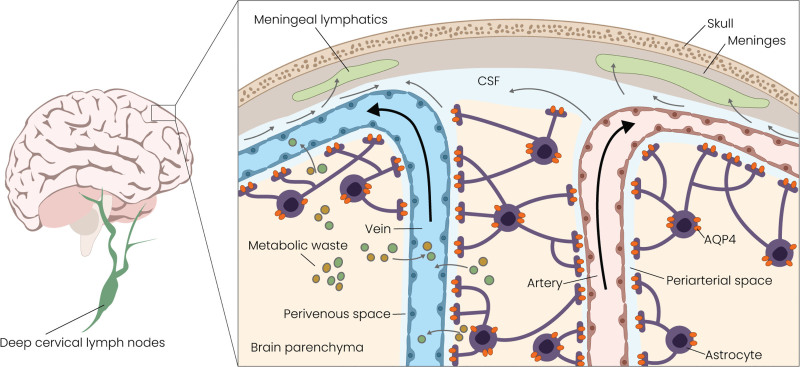
The glymphatic and meningeal lymphatic pathways in central nervous system clearance. Facilitated by astrocytic aquaporin-4 (AQP4) water channels, CSF enters the brain parenchyma, mixes with ISF, and facilitates metabolic waste clearance.

Cervical lymph nodes (cLNs) represent the final checkpoint in the brain lymphatic clearance pathway, acting as an interface between waste originating from the CNS and the peripheral immune system (Fig. 1). Distributed throughout the neck, cLNs receive lymphatic drainage not only from the head and neck but also specifically from the meningeal lymphatics.^16^ Louveau et al^10^ measured a substantial increase in the number of meningeal T cells when dissecting cLNs, indicating an inability of T cells to drain from meningeal spaces once this anatomical connection has been compromised. The cerebral lymphatic efflux pathway is impaired with aging and neurodegeneration^17,18^; cLNs remove metabolic debris and neurotoxic proteins while initiating appropriate immune responses to CNS-derived solutes such as oligomeric α-synuclein and Aβ.^16,19,20^ Interestingly, a recent study in mice highlighted a circadian component to this drainage process. Although glymphatic activity and influx are increased during sleep, especially deep sleep, CSF drainage to the cLNs is higher during wakefulness.^14^

## BRAIN LYMPHATIC IMPAIRMENT AND PATHOGENESIS OF NEUROLOGICAL DISORDERS

Neurological disorders pose a major burden for patients and the healthcare system. Recent research highlights the potential role of impaired lymphatic clearance in their pathogenesis, suggesting that disruptions in the brain’s waste removal systems may contribute to the accumulation of neurotoxic proteins and the progression of chronic inflammation.^1,5,18,21–30^

### Alzheimer Disease

Alzheimer disease (AD) is a neurodegenerative disease characterized by extracellular Aβ plaques and intracellular neurofibrillary tangles of hyperphosphorylated tau protein.^31^ Impaired lymphatic clearance could significantly contribute to the accumulation of these neurotoxic proteins, triggering the chronic neuroinflammation observed in AD. In the healthy brain, extracellular Aβ is eliminated via multiple pathways to prevent downstream effects of Aβ accumulation in the brain.^32^ The glymphatic system drives the convective movement of CSF through perivascular spaces, facilitating exchange with ISF and the removal of soluble Aβ. Once in the CSF, Aβ is transported out of the cranium through meningeal lymphatic vessels, which, ultimately, drain into the deep cLNs and the systemic circulation.^20^

Recent research revealed that the glymphatic system may be compromised in AD.^1,18,21–25^ Iliff et al^1^ proposed that the glymphatic system removes Aβ from the brain via an AQP4-dependent ISF flow. They demonstrated that the clearance rate of radiolabeled Aβ in AQP4-deficient mice was reduced by more than 50% compared with wild-type controls, which might result in the formation of extracellular Aβ aggregates and disease progression in AD.^1^ Further studies using AD murine models have shown that glymphatic transport is markedly suppressed in aged animals, leading to significant Aβ deposition, cerebral amyloid angiopathy, and astrocytic atrophy. Notably, reductions in glymphatic clearance were observed even before the appearance of substantial amyloid plaques in younger mice, suggesting that early impairment of this clearance mechanism may serve as a biomarker for AD.^23,24^ Human studies have also reported impaired glymphatic function in AD.^3,17^ These findings resonate with pathological examinations showing a high prevalence of Aβ in human lymph nodes, with the highest concentrations in the cervical region.^20^ These data are consistent with a model of Aβ drainage to cLNs in humans, although direct evidence on dysfunction of lymphatic clearance in human AD is lacking at this point.

### Parkinson Disease

Parkinson disease (PD) is a progressive neurodegenerative disorder characterized by the loss of dopaminergic neurons in the substantia nigra and the pathological aggregation of misfolded proteins such as α‐synuclein.^33^ Preclinical evidence indicates that the glymphatic system may be impaired in PD.^5,26–28^ Zou et al^34^ showed that ligating the deep cLNs in A53T mice expressing mutant human α‐synuclein led to perivascular clustering of α‐synuclein and disrupted AQP4 polarization in the substantia nigra. This resulted in an increased buildup of α‐synuclein, activation of glial cells, heightened inflammation, loss of dopaminergic neurons, and motor deficits, pointing to the involvement of compromised lymphatic clearance in PD pathology.^27,34^ Zhang et al^5^ demonstrated that deletion of the AQP4 gene resulted in reduced clearance of injected α‐synuclein in mice. Finally, non-REM sleep enhancement in murine PD models reduced α‐synuclein accumulation, which was associated with increased recruitment of AQP4 to perivascular sites, further corroborating the role of increased glymphatic function.

Importantly, clinical studies, particularly with neuroimaging data, have also reported impaired neurolymphatic function in PD.^4,26,28^ Shen et al^4^ showed significantly lower diffusion tensor image analysis along the perivascular space scores—a proxy for glymphatic function—in 76 PD patients compared with a control group. Consistent with the concept of sleep-dependent clearance, a recent study linked impaired deep sleep to magnetic resonance imaging–visible perivascular spaces, another imaging marker of glymphatic dysfunction, in PD patients.^35^

### Traumatic Brain Injury

Li et al^29^ investigated the impact of mild traumatic brain injury (TBI) on the glymphatic system in a rat model. Ten weeks postinjury, contrast-enhanced magnetic resonance imaging revealed significantly reduced clearance rates of a contrast agent and prolonged clearance times in TBI-affected animals compared with healthy controls, indicative of glymphatic dysfunction. These findings were supported by Christensen et al,^36^ who examined glymphatic alterations in a TBI rat model. Clinical trials substantiate these preclinical findings: in a cohort study of 58 patients with mild TBI, Yang et al^30^ reported significantly lower diffusion tensor image analysis along the perivascular space scores compared with 34 healthy controls.

### Intracranial Hemorrhage

Liu et al^37^ investigated the role of AQP4 in hematoma clearance following intracerebral hemorrhage in rat and murine models, respectively, under conditions of regulated AQP4 expression. In their study, dysregulated AQP4 expression was associated with glymphatic dysfunction, neurological impairment, and increased hematoma volume.

## LYMPHATIC MICROSURGERY AS A THERAPEUTIC AVENUE

### Clinical Data

Traditionally, the treatment of the aforementioned neurological conditions has primarily relied on pharmacological approaches. However, the discovery of a mechanical drainage component in their etiopathogenesis has drawn attention toward lymphatic microsurgical interventions, particularly cervical LVA.^38^ This surgery connects lymphatic vessels and venous structures, thereby bypassing obstructed or dysfunctional lymphatic pathways. Previous studies have demonstrated the technical feasibility and effectiveness of LVA in treating both upper and lower limb lymphedema.^39–41^ By enhancing lymphatic fluid flow from the CNS, LVA may alleviate glymphatic dysfunction, reduce protein aggregation, and potentially slow or reverse neurodegeneration.

In 2022, Lu et al^9^ published the first clinical case report on deep cervical LVA for the treatment of cognitive dysfunction. It was also reported that Dr. Xie had successfully performed 50 cases of such cervical LVA reconstructions in AD patients, with an average follow-up of 9 months. More recently, Li et al^8^ published another case report describing a supermicrosurgical technique to restore cerebral lymphatic drainage in a patient with AD. The aim of this approach was to enhance the clearance of proteins such as Aβ and tau from the brain’s lymphatic system into the cervical lymphatics, thereby reducing toxic protein accumulation in the brain.

Although the pioneering work from China has laid a strong foundation, extensive preclinical studies and well-designed clinical trials are warranted. Several trials are underway to explore lymphatic supermicrosurgery in neurological disorders. Multiple prospective clinical trials investigating the effect of cervical LVAs in AD have been registered (NCT06448442, NCT06448975, ChiCTR2500095309, ChiCTR2400093030 [AD and type 2 diabetes], ChiCTR2400089883, ChiCTR2400094603, and ChiCTR2400084617).

Yang et al^7^ have extrapolated the preliminary yet promising findings from the field of AD to hypothesize that deep cervical LVA could also be leveraged for PD treatment. To date, however, there are no known clinical case reports in which cervical LVA has been performed for the treatment of PD. Likewise, despite the aforementioned etiopathogenetic links between neurolymphatic impairment and further CNS disorders, including TBI or intracerebral hemorrhage, no human studies or clinical cases involving LVA have been documented in these contexts. A prospective study has been launched investigating cervical LVAs in patients with PD (ChiCTR2400091857). To our knowledge, no studies have yet been planned/registered on the surgical treatment of other neurological disorders via lymphatic microsurgery.

Numerous ongoing investigations in this field highlight the growing research momentum while emphasizing the pressing need for high-quality empirical data. A thorough preoperative assessment of the patient’s lymphatic function would, therefore, be essential to determine patient eligibility for the procedure. Histological evidence of lymphatic vessels could not be identified in the brain parenchyma, in part because of postmortem changes in the tissue. The blood-brain barrier was believed to prevent the diffusion of molecular and cellular components. Further research with objective, high-quality outcome studies remains indispensable to investigate both the safety and efficacy of LVA for the treatment of neurological disorders. Special consideration should be given to ethical concerns when planning clinical studies involving patients with neurodegenerative diseases or debilitating cranial injury, as these individuals represent a vulnerable patient population. Beyond the general ethical obligations inherent to surgical innovation, the application of lymphatic microsurgery in neurological disorders poses specific challenges. Informed consent must be carefully tailored to account for potential cognitive impairment, and surrogate decision-making may be necessary in select cases. Transparent communication of the experimental nature of the intervention, as well as the lack of large-scale efficacy data, is essential to avoid therapeutic misconception. Furthermore, equitable access to such advanced surgical options—including those involving costly robotic assistance—must be proactively addressed to prevent widening disparities in neurological care.

### Robotic-assisted Lymphatic Microsurgery

**Fig. 2.**
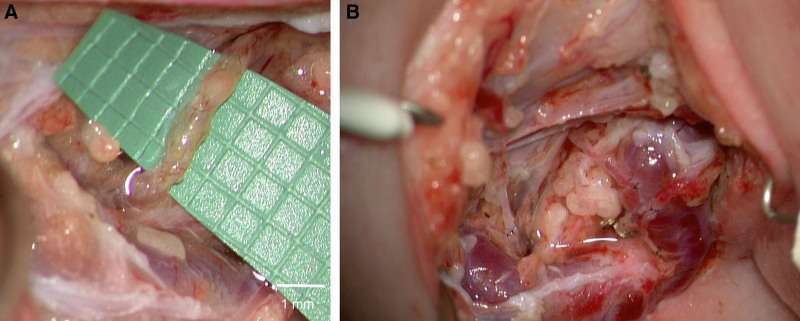
Deep cervical LVAs. A, Thoracic duct in the deep left neck (0.9-mm diameter) of a 6-month-old child with severe central lymphatic flow disorder with chylothorax, chylaskos, protein-losing enteropathy, and head and neck lymphedema. B, (1) LVA of thoracic duct anterograde end-to-side to collateral external jugular vein, (2) LVA of thoracic duct retrograde end-to-end to collateral vein, and (3) multiple LVAs of deep lymphatic collectors draining the head in the left neck.

Given the small diameter of many lymphatic vessels (<0.5 mm), LVAs require high-magnification microscopes and specialized microsurgical instrumentation. The use of robotic systems, such as the MUSA robot (MicroSure B.V., Son, The Netherlands) and the Symani Surgical System (Medical Microinstruments [MMI], Jacksonville, FL), is well established in the surgical treatment of lymphedema and central lymphatic lesions.^42–46^ Our team documented the first-in-human use of a microsurgical robotic system for central lymphatic reconstruction in 2023.^47^ Since then, we have successfully treated patients presenting with thoracic duct lesions or thrombotic occlusions via LVA in the deep neck, further solidifying our expertise in this fast-evolving field (Fig. 2).^48–50^ (See Video [online], which displays an 8-month-old patient who had a history of severe bilateral chylothorax and protein-losing enteropathy due to thrombotic occlusion at the left venous angle. Robotic-assisted end-to-end anastomosis of the thoracic duct (0.9 mm) with a branch of the left external jugular vein was performed in the deep neck.)

**Video 1.** The 8-month-old patient had a history of severe bilateral chylothorax, protein-losing enteropathy due a thrombotic occlusion at the left venous angle. Robotic-assisted end-to-end anastomosis of the thoracic duct (0.9 mm) and a branch of the left external jugular vein was performed in the deep neck. Reproduced under a CCBY-NC-ND license (https://creativecommons.org/licenses/by-nc-nd/4.0/), the figure legend has been modified.50

Recently, we shared our experience with 100 (super-)microsurgical anastomoses in both peripheral and central lymphatic reconstruction performed with robotic Symani assistance.^51^ Postoperatively, no anastomosis-related complications were observed, all anastomoses remained patent, and most patients reported postoperative improvement. These promising findings on robotic-assisted LVA are echoed on a global level.^52^ Accordingly, the scientific community increasingly recognizes the potential benefits of robotic surgical systems with possible applications extending to cervical LVAs. Robotic systems provide better access to deep anatomical planes and small structures, facilitating precise dissection and suturing.^53^ The integration of wristed robotic arms improves the range of motion compared with conventional microsurgical procedures.^53–55^ Motion scaling and tremor elimination are crucial in improving surgical accuracy by filtering out unintended hand tremors and translating the surgeon’s movements with greater accuracy.^53,54^ Conventional microsurgery typically requires surgeons to maintain a prolonged static posture, negatively affecting performance over time.^56,57^ Robotic platforms offer ergonomically optimized working environments, potentially minimizing fatigue and physical strain and enhancing long-term surgical accuracy.^56,57^ Finally, the use of robotic platforms may offer improved visualization through advanced imaging technologies.^53^ High-definition 3-dimensional magnification, combined with augmented reality overlays and digital enhancements, allows for superior differentiation of tissue planes and microvascular structures.^58^

Despite its immense potential, several challenges must be overcome before robotic-assisted lymphatic supermicrosurgery can be widely adopted and integrated into the future surgical treatment of neurological disorders via cervical LVA. The acquisition and maintenance costs of robotic systems remain high, yet increasing acceptance and technological dissemination could lower costs over time.^53,59^ In addition, the effective and safe use of robotic platforms in lymphatic surgery requires specialized training. Surgeons must complete structured educational programs to develop proficiency in robotic-assisted procedures.^60^ However, it is important to note that, although initial operative times may be longer, the steep learning curve leads to significant improvements in efficiency.^61^ The rigidity of robotic arms can limit the range of motion and flexibility and restrict access to certain surgical sites. Accordingly, careful preoperative planning and the establishment of optimal access routes are essential for successful procedure execution. Our experience suggests that surgeons can adapt to the missing sensory feedback in robotic systems, as the force required to manipulate the robotic system is perceived as equivalent to that needed for conventional microsurgical instruments.^45^

The convergence of these 2 emerging fields—robotic supermicrosurgery and lymphatic reconstruction for the management of neurological disorders—holds immense potential for advancing treatment strategies and redefining therapeutic paradigms. Robotic assistance enables greater precision in manipulating delicate lymphatic structures, improving the efficacy of lymphatic reconstruction not only in the extremities but also potentially in the cervical region. In this context, it is also worth noting that if surgical intervention on the glymphatic system were ever to be performed, the use of robotic assistance would be practically mandatory due to its minuscule size of less than 100 µm.^62^ Further research is essential to bridge these disciplines and their synergistic advantages into clinically meaningful outcomes.

## CONCLUSIONS

The discovery of the brain’s lymphatic system has revolutionized our understanding of CSF homeostasis and neuroimmune regulation, providing novel insights into the pathophysiology of neurological disorders. Impaired glymphatic and meningeal lymphatic drainage is increasingly recognized as a major factor in the accumulation of neurotoxic proteins, neuroinflammation, and progressive neuronal dysfunction. In this context, lymphatic supermicrosurgery—particularly deep cervical LVA—has emerged as a promising intervention to enhance CNS lymphatic clearance and facilitate the removal of toxic waste products from the brain. Early clinical reports suggested that LVA is not only technically feasible but may also improve cognitive function in select patients with AD.

Despite these encouraging preliminary findings, thorough scientific validation remains imperative. The available evidence is limited to case reports, and further clinical studies are needed to elucidate the precise mechanistic impact of LVA on CNS fluid dynamics and its long(er)-term therapeutic potential. Future research should also focus on refining patient selection criteria, maximizing surgical safety—potentially incorporating robotic-assisted microsurgery—and exploring synergistic therapeutic strategies, including pharmacological modulation of lymphatic function. Given the nascent stage of this field, this article synthesizes early clinical insights with emerging experimental evidence and distinguishes between hypothesis and data, aiming to provide a structured, scientifically grounded framework for future investigation. To minimize potential bias and avoid overinterpretation, we have contextualized subjective observations and highlighted the need for robust, controlled, and objective studies moving forward. Ultimately, widespread clinical adoption will depend on the generation of high-quality, objective evidence from well-designed trials. However, unlocking the full potential of lymphatic supermicrosurgery is not merely a surgical challenge but a watershed moment in redefining brain health itself. If successful, LVA could herald a new era—one in which restoring the brain’s intrinsic clearance mechanisms becomes a cornerstone of neurological disorder therapy, offering hope where there are currently few options.

## DISCLOSURE

Dr. Lindenblatt acts as a consultant and scientific advisor for Medical Microinstruments (MMI). The other authors have no financial interest to declare in relation to the content of this article.

## PATIENT CONSENT

All patients have given written permission to publish the intraoperative images and signed the hospital’s general consent form.
